# Improvement of wet rub fastness in continuous dyeing with c.i. Sulphur Black 1 by ultrasonic treatment

**DOI:** 10.1016/j.ultsonch.2023.106558

**Published:** 2023-08-14

**Authors:** Margit Lenninger, Thomas Bechtold, Tung Pham

**Affiliations:** Universität Innsbruck, Research Institute of Textile Chemistry and Textile Physics, Hoechsterstrasse 73, 6850 Dornbirn, Austria; Universität Innsbruck, Innrain 52, 6020 Innsbruck, Austria

**Keywords:** C.I. Sulphur Black 1, Ultrasonic treatment, Colour fastness, Pad-steam dyeing, Sulphur dyeing

## Abstract

C.I. Sulphur Black 1 is among the most important dyes for cellulose textiles, as this dye offers a maximum colour depth at reasonable costs. In its final form the dye is present as solid pigment, thus limited wet rub fastness can be an issue. Controlled re-oxidation of the leuco form into the oxidised dye pigment and intensive washing and soaping are measures to achieve acceptable wet rub fastness. In this study pad-steam dyeing with C.I. Sulphur Black 1 was used to investigate the effects of an intensified washing process with use of ultrasonification on rub fastness. Ultrasound was applied following to steam fixation of the dye. Introduction of ultrasound into the following process steps was investigated: water seal, warm rinse, oxidation, soaping, cold rinse. Best results in rub fastness were obtained by combination of soaping and ultrasound processing. The high shear rate near to the fibre surface led to a reduction of the thickness of the stagnant diffusion layer and more intense removal of loosely bound dispersed dye. As a result of the treatment an improvement in wet rub fastness of the dyeings was achieved. Duration of 60 – 120 s was found sufficient to remove the major part of surface bound pigments which allows an integration into continuous pad-steam dyeing processing. The results demonstrate that ultrasound washing may lead to improved rub fastness of other dyeings where a pigment is the final form of the dye. The method thus could be extended to vat dyes including indigo, or naphthol dyes.

## Highlights

• Ultrasound treatment improves fastness of sulphur dyeings.
• In after-treatment combination of ultrasound treatment and soaping is most effective.
• Improvement of wet rub fastness of C.I. Sulphur Black 1 about 1 grade was achieved.
• Short treatment for 60 – 120 s allows integration in continuous dyeing.

## Graphical abstract

**Figure:**
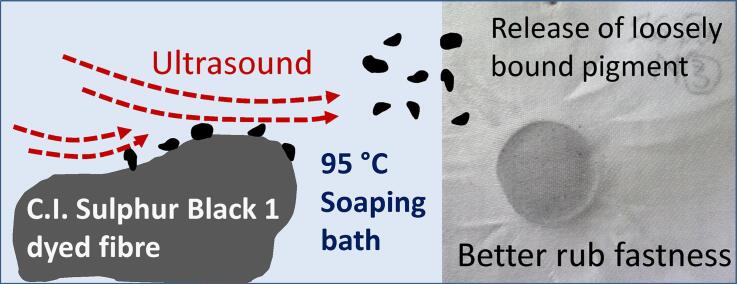

## 1 Introduction

With an estimated annual production in the dimension of 100,000 tons C.I. Sulphur Black 1 belongs to the most important dyes for cotton and cellulosics [1]. Sulphur dyes offer a maximum colour depth with good fastness for a comparable low price [2]. During application sulphur dyes are transferred into their reduced leuco form which sorbs on the cellulose fibre and then becomes re-oxidised to the dye pigment [3]. During re-oxidation of the leuco dye a small amount of dispersed dye pigments deposits on the surface of the textile fibres. These loosely bound pigments become released under frictional forces which leads to limited rub fastness particularly in wet state [4]. More complete removal of these loosely attached dye particles will contribute to an improved rub fastness in dry and wet state.

The removal of water soluble dyes e.g. hydrolysed reactive dyes require desorption of the molecules from the fibre and diffusion through the stagnant diffusion layer between fibre surface and bulk solution. Increased temperature and mechanical action support a more rapid equilibration between substrate and rinsing bath.

In case of solid particles intensive mechanical agitation supports the release of loosely bound parts [5]. Ultrasound (US) processes have been studied extensively as technique to intensify the mechanics in laundry for better soil removal [6], [7], [8]. Introduction of US led to a substantial intensification of the wash intensity and more complete soil removal with low energy consumption [9], [10], [11]. Also localised pre-treatment of contaminated textiles with handheld US generators and use of a detergent solution has been reported to improve stain removal during the following wash cycle [12].

The promising potential of US processes to intensify transport processes at the interface between textile fibre and processing bath has led to a number of studies to elucidate the potential of the technique in textile chemical processes [13]. US treatment has been used to intensify dye removal and demineralisation in pre-treatment processes [14], [15]. The intensification of bath exchange and dye penetration in dyeing processes with US has been studied in reactive dyeing [16], [17]. Therein higher production efficiency and reduced energy consumption has been achieved by assistance of US treatment [18], [19].

US processes have led to a reduction of particle size of dispersed vat dye and to an increased reduction rate in vatting with use of organic reducing agents [20], [21]. Positive effects on colour depth and fastness have been reported from a combination of US treatment and exhaust dyeing with vat dyes [22]. In polyester dyeing and rinsing of printed goods beneficial effects also have been observed from the intensified exchange between textile goods and treatment bath [23], [24].

A number of studies has investigated assistance of US for intensification of dye sorption on solid adsorbents [25], [26].

The majority of studies available in the literature address the use of US during the dyeing process to intensify the exchange between dyebath. Beneficial effects also have been reported for the application of US during the reductive cleaning of poly(lactic acid) fibres, which had been dyed with disperse dyes [27], [28].

To the best of our knowledge the specific potential of US processes to remove loosely bound finely dispersed dye particles has not been studied up now. In particular in sulphur dyeing an US treatment could improve the wet rub fastness of dyed material and thereby improve its quality level substantially. In this study continuous pad-steam dyeing with C.I. Sulphur Black 1 has been chosen as a reference process to answer two research questions: Will an integration of an US treatment improve the rub fastness of the dyeings and at which stage of the continuous dyeing process an US treatment will yield the best results?

In the experimental work laboratory scale pad-steam dyeing with C.I. Sulphur Black 1 was combined with systematic work to identify the process step in the after-treatment where US treatment is most effective. The duration of the treatment was kept relatively short to permit possible integration into continuous pad-steam dyeing. The dyeings were characterised by their CIELab colour coordinates and the dry and wet rub fastness. Optimised conditions then were used to run a continuous pad-steam dyeing in lab scale with an integration of US treatment at the stage of soaping to demonstrate the new concept.

## 2 Experimental

### 2.1 Materials and chemicals

For the experiments a fabric (plain weave, 120 g m^−2^) made of 50 %wt cotton, 50 %wt PET fibre was used. The polyester fibres had been pre-dyed black with disperse dyes in a continuous pad/dry/thermosol fixation process.

For the dyeing experiments technical grade chemicals were used (aqueous solution of NaOH 32.5 %wt; acetic acid 80 %wt; H_2_O_2_ 35 %wt).

**Fig. 1:**
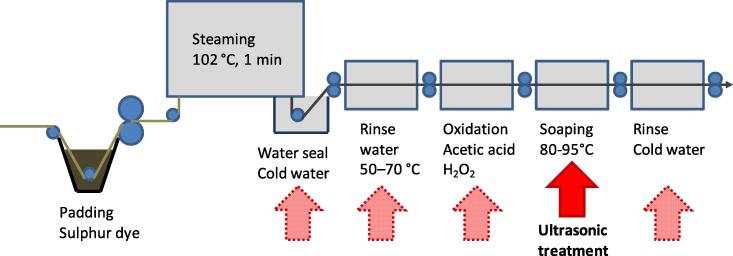
General process scheme of the pad-steam sulphur dyeing; red arrows indicate positions where integration of US treatment was studied.

For the sulphur black dyeing a pre-reduced formulation of C.I. Sulphur Black 1 (Diresul Black RDT-LS 200%, Clariant, Switzerland), a sequestering agent (Dekol SN, BASF Ludwigshafen, Germany) and organic carbohydrate based reducing agent (Reductor D, Clariant, Switzerland) were used. For soaping of the dyed samples surfactant (Sandopur RSK, Clariant, Switzerland) was used. The detailed recipes are given below along with the description of the respective dyeing process (Table 1, Fig. 1).

**Table 1:** Composition of dye solution in pad-steam dyeing. Series A – D.

Component	Series	A	B	C	D
Padding					
Dye (Diresul RDT-LS 200%)	g L^-1^	221	145	157	155
Dekol SN	ml L^-1^	3	3	3	3
Reducing agent	g L^-1^	25	25	25	25
NaOH 32.5 %wt, 38°Bé	ml L^-1^	20	23.6	20	22.6

Oxidation					
Acetic acid 80 %	ml L^-1^	2.5	2.5	2.5	2.5
H_2_O_2_ 35 %wt	ml L^-1^	8	8	8	8

Soaping					
Sandopur RSK	g L^-1^	2	2	2	2
Na_2_CO_3_	g L^-1^	2	2	2	2

### 2.2 Dyeing process

The dyeing experiments were performed as pad-steam dyeings on a laboratory scale continuous dyeing unit (ca. 20 cm fabric width, Ernst Benz AG, Rümlang Zürich, Switzerland). A general process scheme is given in Fig. 1. A photo of the machine is given in the Supplementary Material Figure S1. The dry material was first padded with a dye solution (145–––220 g/L Diresul Black RDT-LS 200 %, 20 – 22 ml/L NaOH, 32.5 %wt, 25 g/L Reductor D, 3 g/L Dekol SN; pressure 1.8 bar, 3 m min^−1^, pick up 80 %wt). The material then was steamed for 1 min at 102 °C. After a first short rinse in the water seal at the end of the steamer the samples were rinsed at 50 – 70 °C and oxidised with a solution of acetic acid and hydrogen peroxide (50 °C, 2 min). The samples then were soaped in a solution of surfactant (Sandopur RSK) and soda at 80 °C or 95 °C and finally rinsed in cold water.

### 2.3 Ultrasonic treatment

A commercial US bath with 20 L bath volume operating at 35 kHz (300 W ultasonic power) was used for the experiments (Transsonic 890/H, Zeller GmbH, Hohenems, Austria). At first material samples were collected after a certain stage of the continuous dyeing process. In the following step a combination of after-treatment and US processing was applied for a defined time e.g. peroxide oxidation and ultrasonification for 60 s. At the end of the US treatment each sample then was processed further according the process scheme in Fig. 1. In the continuous dyeing with US treatment the material was introduced into the US bath in rope form. The length of the fabric rope in the US bath was adjusted to realise 60 s of US treatment.

### 2.4 Colour measurement and fastness testing

The CIELab colour coordinates of the dyed samples were determined in four layers with a tristimulus colorimeter (Minolta CR-210, 50 mm sample diameter, measurement geometry d/0°). All CIELab coordinates were calculated for light source D65. The L* coordinate describes the lightness of the sample (white L* = 100, black L* = 0), the a* coordinate describes the position on the red-green axis (red = positive a*, green = negative a*), the b* coordinate describes the position on the yellow-blue axis (yellow = positive b*, blue = negative b*).

Fastness to rubbing was tested with a crockmeter according DIN 54021 [29]. The colour change of the white cotton fabric used in the rub fastness testing was measured with use of a smaller sample diameter (Minolta CR-200, 8 mm sample diameter, measurement geometry d/0°). Staining of the white fabric used for rubbing was assessed with colour measurement and with use of the standard grey scale (grading 5 for excellent fastness and 1 poor fastness). Results are given as mean and standard deviation of three repetitions.

## 3 Results and discussion

### 3.1 Identification of optimum position for US-treatment

A general process scheme of the continuous pad-steam dyeing is given in Fig. 1. While there is no doubt about the relevance of a thorough washing and soaping of a dark sulphur dyeing the correct site for removal of loosely bound dispersed dye particles still is under discussion [4]. The optimum positioning of the US treatment was investigated by a series of experiments in which material samples were collected at different process steps and treated with US (series A). The samples then were finished in batch treatment according the conditions given in Table 1.

A lab scale continuous pad-steam dyeing was executed to identify the process step where an US treatment would be most effective (Series A). Following to steam fixation of the sulphur dye an US treatment could be applied at different process stages: during the passage through the water seal, during the warm rinse before oxidation, during peroxide oxidation, in the soaping step or during the final cold rinse. Samples were taken at the respective stage and treated with US for 60 s. Then the samples were finished using the standard procedure. The length of the US treatment in the batch tests was defined with 60 s to permit later integration of the technique into continuous pad-steam dyeing. At a production speed of 30 m min^−1^ an US treatment for 60 s already requires a capacity of 30 m fabric in the respective section of the plant.

**Fig. 2:**
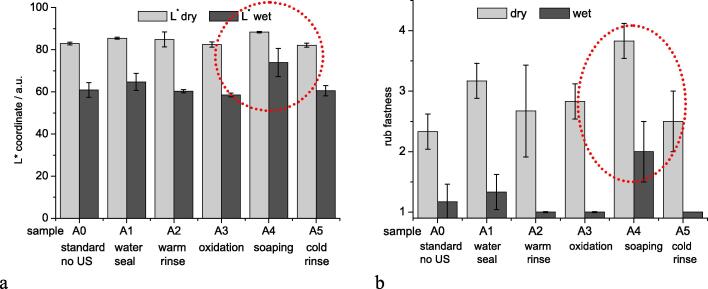
Identification of optimum position of US treatment in a continuous dyeing process. Effect of position of US processing during after-treatment on rub fastness in dry and wet state. a) L*-coordinates of white fabric used in rub fastness test (Diagram including a* and b* coordinates given in Supplementary Information, Figure S2); b) ratings from dry and wet rub fastness testing with use of grey scale; error bars represent standard deviation of three repetitions.

The results given in Fig. 2 indicate that a combination of the US treatment with the soaping process leads to the most effective increase in rub fastness of the dyed fabric both in dry and wet state. The highest L* value of the white fabric used for rubbing indicates the lightest staining during dry and wet rub testing (Fig. 2a). By combination of US treatment and soaping an improvement of the wet rub fastness of nearly one mark of the grey scale rating is achieved (Fig. 2b). Colour measurement of the dyeings indicated, that no adverse effect on the product colour resulted from the different treatments (Supplementary Information Table S1).

### 3.2 Optimisation of US-treatment

As a consequence the optimisation of US treatment during the soaping was studied in more detail (Series B). Specimens were soaped at 80 °C or 95 °C and the effects of US treatment (60 s) and addition of surfactant (2 g/L Sandopur RSK, 2 g/L Na_2_CO_3_) were studied. In Table 2 the experimental conditions applied in Series B are shown. A sample without any soaping (B0) was collected as a reference to assess the improvement in fastness by any treatment. Colour measurement of the dyeings indicated, that no adverse effect on the product colour resulted from the different treatment. The colour coordinates of the dyeings are given in the Supplementary Information (Table S2).

**Table 2:** Experimental conditions used to optimise the combination of US treatment and soaping (Results given in Fig. 3).

Sample	Temp.	Surf	US
	°C		
B0	–	–	–
B1	80	+	–
B2	80	–	+
B3	80	+	+

B4	95	–	–
B5	95	+	–
B6	95	–	+
B7	95	+	+

**Fig. 3:**
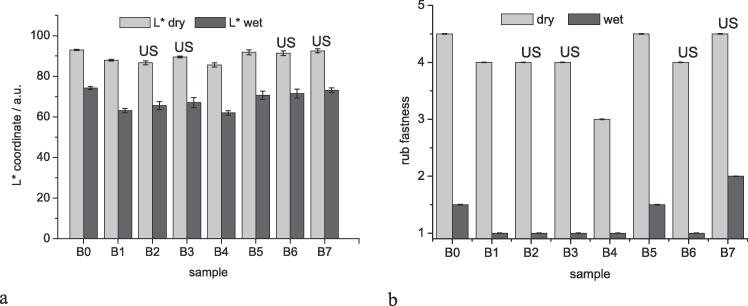
Effect of temperature, surfactant and US treatment (US) at the stage of soaping on rub fastness on a) L* coordinates of the white test fabric used (Diagram including a* and b* coordinates given in Supplementary Information, Figure S3); b) dry and wet rub fastness of the dyeing using the grey scale rating; error bar represents standard deviation of three repetitions.

The influence of the different processing on the colour fastness is shown in Fig. 3. Good rub fastness in dry state (4 and 4–5) was obtained for almost all samples. Differences in wet fastness were dependent on the particular conditions applied. The results given in Fig. 3 demonstrate that highest rub fastness (4–5 in dry state, 2 in wet state) could be achieved with combination of soaping at 95 °C, use of surfactants and US treatment (B7). Second best wet rub fastness was observed for samples with soaping at 95 °C and use of surfactants (B6). A similar rating was observed for the sample without any soaping (B0), which however requires further analysis. The CIELab colour coordinates of the treated samples are given in the Supplementary Information Table S2.

In the next series of experiments the required duration of US treatment to remove the majority of loosely bound pigments was studied (Series C). Samples were collected after the stage of oxidation and treated with US for different time.

**Fig. 4:**
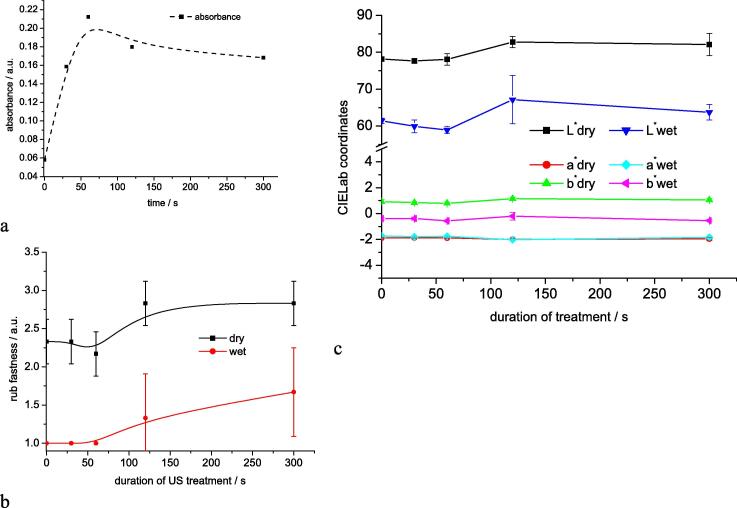
Release of dye pigment and change of rub fastness with duration of US treatment (series C); a) absorbance of reduced dye as function of time; b) improvement of rub fastness with duration of US treatment in terms of CIELab coordinates of the white fabric used for rub fastness testing; c) dry and wet rub fastness of the dyeings; fastness values given as mean and standard deviation of three repetitions.

To quantify the release of sulphur pigment into the soaping bath solution the dispersed dye was transferred into its reduced form by addition of 2 ml NaOH 38Bé and 0.8 g Na_2_S_2_O_4_ to 100 ml of dispersion. The absorption of the solution then was analysed at 425 nm and used as relative measure for the pigment concentration in the bath. The release of dye pigment with duration of US treatment is shown in Fig. 4a. The results given in Fig. 4a indicate that a 60 s US treatment already is sufficient to remove the major part of loosely adhering dye pigment. The rub fastness of the samples in terms of CIELab coordinates of the white fabric used for rubbing and the assessment with use of the grey scale are given in Fig. 4b and c. The CIELab colour coordinates of the treated samples are given in the Supplementary Information (Table S3). Under the conditions of the experiments an increase in lightness of the white test fabric (Fig. 4b) and an improvement of the rub fastness (Fig. 4c) was observed after 120 s of US treatment. No further improvement was observed with longer treatment for 300 s. Duration of 60–120 s for the US treatment makes the process applicable in a continuous pad-steam dyeing. As the size of a compartment will increase with the resident time required for US treatment longer treatment would lead to in-acceptably large technical installations.

### 3.3 Continuous pad-steam dyeing with US-treatment

The reproducibility of the results from series A - C and the integration of the US-treatment into a simulated continuous operation then was tested in a modified laboratory pad-steam process (series D). The pad-steam dyeing of the fabric was performed in open-width form according the scheme in Fig. 1. At the stage of combined soaping and US treatment the fabric was processed in rope form for 60 s, followed by a rinse in cold water.

**Fig. 5:**
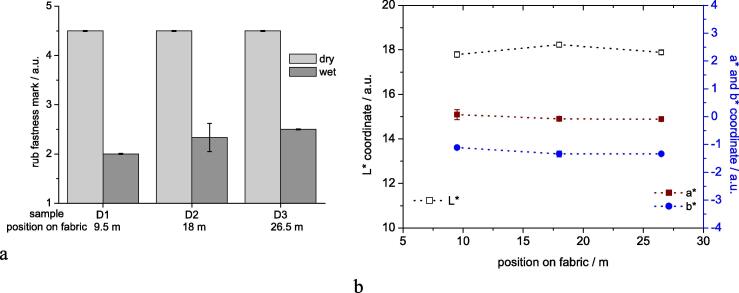
Combination of continuous pad-steam dyeing and US-treatment during soaping; a) rub fastness dry and wet (marks according grey scale), b) CIELab coordinates of the dyeings as function of processed length of sample.

A total length of 27 m of fabric was processed at a speed of 3 m min^−1^ which corresponds to a total duration of 9 min. Samples of the processed fabric were taken at a production length of 9.5 m (3.2 min), 18 m (6 min) and 26.5 m (8.8 min) and tested for rub fastness and CIELab colour coordinates (Fig. 5a and b).

In the continuous dyeing a level of 2 – 2.5 could be achieved for the rub fastness in wet state which is in agreement with results from previous experiments (Series A – C). The dry rub fastness remained at a stable level 4–5. The shade of the dyeing in terms of CIELab coordinates remained constant along the length of the 2 m long piece of fabric (Fig. 5b).

### 3.4 Discussion of physical–chemical background

**Fig. 6:**
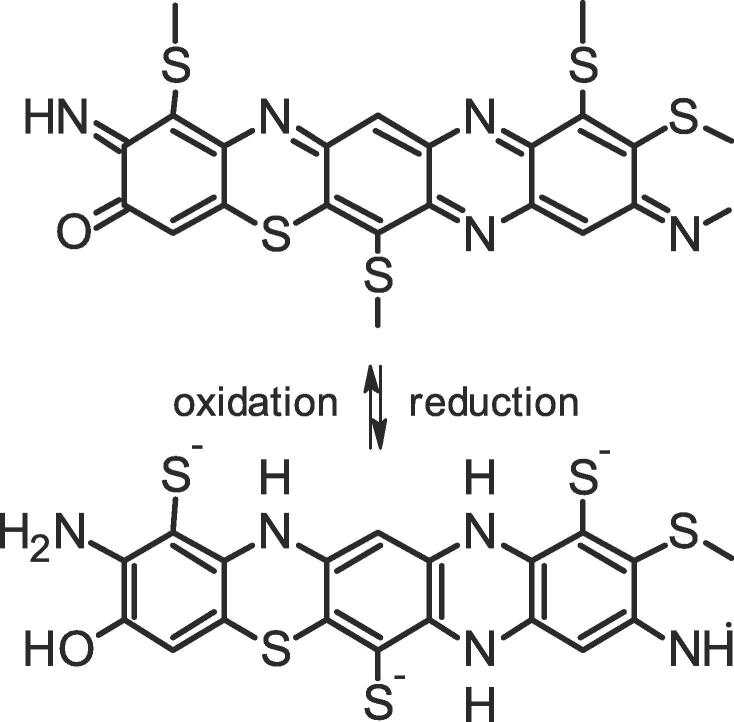
Reduction and re-oxidation of C.I. Sulphur Black 1 during application [30].

The results from the simulated pad-steam dyeing experiments indicate the soaping step as process stage where an US treatment will be most efficient to improve rub fastness. This can be explained on the basis of the physical–chemical processes occurring in the dyeing process (Fig. 1). As condition for dye fixation the sulphur dyes are reduced into their soluble leuco-form and cleavage of disulphide bridges leads to formation of lower molecular weight fragments of the leuco-dye (Fig. 6) [30].

During the steam treatment the leuco-dye diffuses into the cellulose fibre and adsorbs on the cellulose structure. In the cold water seal excess reducing chemicals are removed and also unfixed soluble dye is removed. In the following steps of the continuous dyeing process the oxidation of the leuco-dye into its insoluble pigment form begins. These steps are determining for the final rub fastness as deposits of the oxidised dye will be rubbed off by abrasive forces and thus are the origin for low rub fastness. The formation of the dye pigment completes during the stages of warm rinse and oxidation step, thus effectiveness of the US processing still is limited. The most efficient removal of adherent dye particles is achieved by US processing during the hot soaping step. At this stage the dye has been oxidised completely and the final state of the particles has been formed. In addition the effectiveness of the US treatment is supported by addition of surfactants treatment and elevated temperature. Application of US in the last cold water rinse thus will not yield comparable results.

**Fig. 7:**
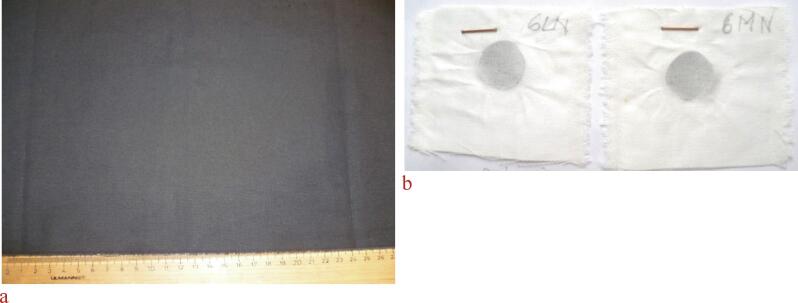
A) photograph of a C.I. Sulphur Black dyeing, b) photograph of a representative test fabrics after determination of rub fastness (the staining at the site of the dark spot defines the rub fastness).

No difference between black dyeings with different rub fastness can be detected visually. Only a very minor share of the overall dye content is deposited on the fabric surface and becomes responsible for a lowered rub fastness. In Fig. 7a photograph of a dyed sample and in Fig. 7b a photograph of test fabrics used for assessment of rub fastness are given.

The application of US processes will improve rub fastness of dyeings most effectively at a process stage where the final pigment form has been formed completely. In case of vat dyeing, indigo dyeing and naphthol dyeing this step will be at the stage of soaping during the after treatment of the dyeings.

## 4 Conclusions

C.I. Sulphur Black 1 is among the most important textile dyes for black shades. The application includes reduction of the water insoluble dye into its alkali soluble leuco form. Following to the sorption phase the leuco dye is re-oxidised into the insoluble dye pigment. The wet rub fastness of such dyeings is limited through deposition of low sized dye particles on the fibre surface, which are removed partly during the hot soaping process.

The high mechanical agitation of an US treatment led to a more intensive removal of such dye deposits and thus contributed to an improved wet rub fastness of C.I. Sulphur Black 1 dyeings. Experiments with continuous pad-steam dyeings showed that the US treatment is most efficient when it is applied in combination with the soaping step. Duration of 60 – 120 s of US treatment is sufficient to increase the wet rub fastness for about one unit. The measurement of the CIELab coordinates of the dyeings did not indicate adverse effects of the US treatment on the colour of the dyed fabric.

The short time required for the US treatment permits an integration of the technique into continuous dyeing plants as the content of such a unit will remain acceptable e.g. with 30 – 60 m of fabric at a production speed of 30 m min^−1^.

The results demonstrate that more intense agitation during after-treatment of sulphur dyeings on cellulose contributes to improved removal of deposited dye pigments and thus improves the final rub fastness of the dyeing. Similar results can be expected for other classes of dyes in which the dye finally is present as an insoluble pigment e.g. vat dyes including indigo as well as naphthol dyes.

## Funding

Financial support is gratefully acknowledged to the COMET Project “Textile Competence Center Vorarlberg 2 – FFG 882502”, funded within COMET – Competence Centers for Excellent Technologies – by BMK, BMDW as well as co-financing federal province Vorarlberg. The COMET-Funding Program is managed by the Austrian Research Promotion Agency FFG.

## CRediT authorship contribution statement

**Margit Lenninger:** Writing – review & editing. **Thomas Bechtold:** Conceptualization, Data curation, Writing – review & editing. **Tung Pham:** Supervision, Funding acquisition.

## Declaration of Competing Interest

The authors declare that they have no known competing financial interests or personal relationships that could have appeared to influence the work reported in this paper.
