# Supplementary material for: Improvement of wet rub fastness in continuous dyeing with c.i. Sulphur Black 1 by ultrasonic treatment
Source: Ultrason Sonochem. 2023 Aug 14;99:106558. doi: 10.1016/j.ultsonch.2023.106558 (PMC10484801; doi:10.1016/j.ultsonch.2023.106558)
Supplement: Supplementary data 1 [file mmc1.docx]

Supplementary Information

**Improvement of wet rub fastness in continuous dyeing with C.I. Sulphur Black 1 by ultrasonic treatment**


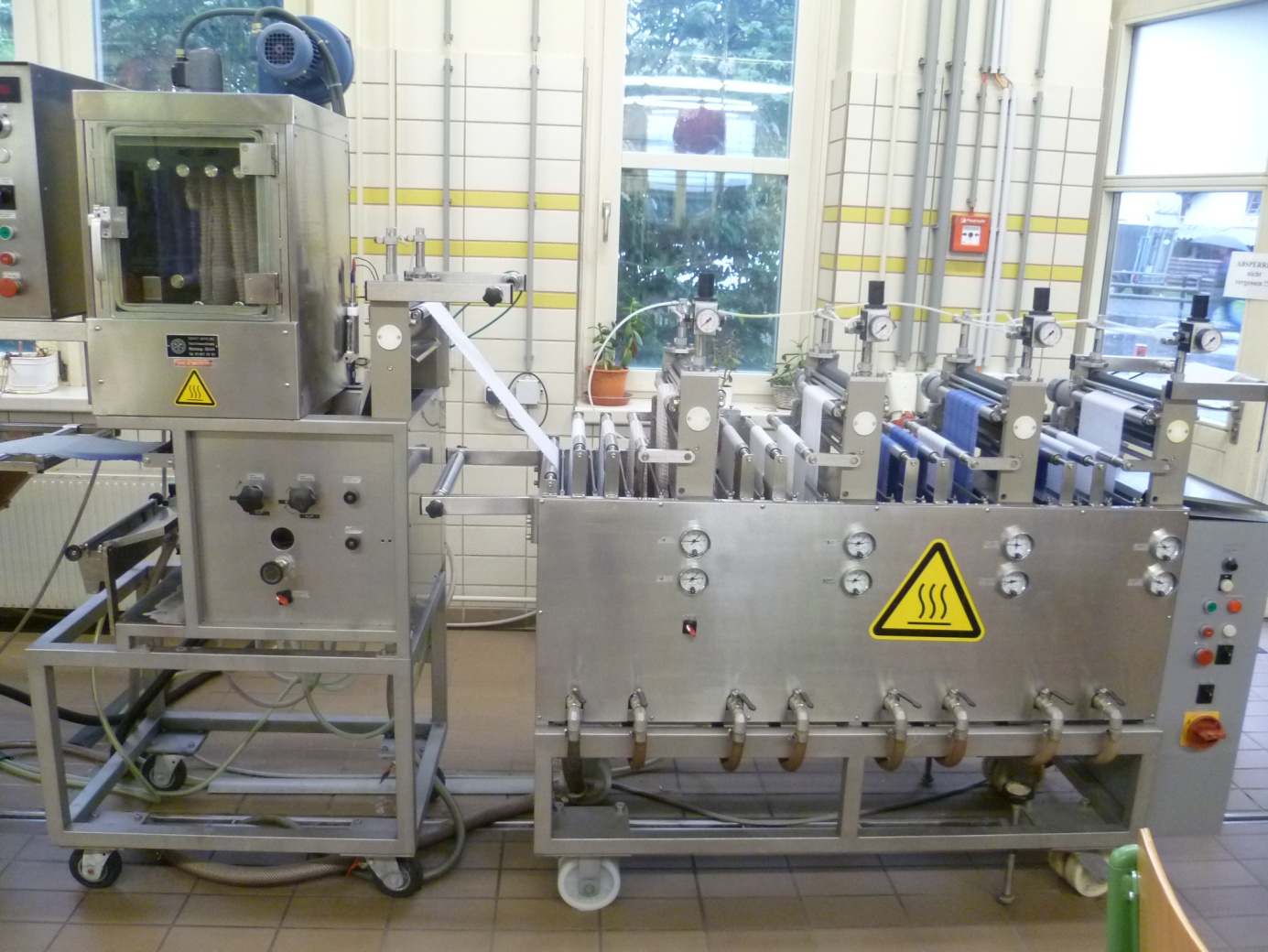


Water seal

Cold rinse

Oxidation

Soaping

Warm rinse

Steamer

**Figure 1.** Photographs of the laboratory pad-steam unit filled with lead-in fabric.

**Figure S2.** Identification of optimum position of ultrasonic treatment in a continuous dyeing process. Effect of position of ultrasound treatment after steam fixation on rub fastness in dry and wet state. a) L*, a* and b*-coordinates of white fabric used in rub fastness test.

**Figure S3.** Effect of temperature, surfactant and ultrasonic treatment at the stage of soaping on rub fastness L*, a*, b* coordinates of the white test fabric used in rub fastness test.

**Table S1**. Influence of position of US treatment in the after-treatment of pad steam dyed fabric in colour of the dyeings in terms of CIELab coordinates and total colour difference ΔE between untreated reference and treated sample (US = ultrasonic treatment, duration of treatment 60 s).

| Sample | Position | Colour coordinate | | | ΔE |
| --- | --- | --- | --- | --- | --- |
|  |  | L* | a* | b* |  |
| A0 | no US | 17.95 | 0.40 | -0.09 | 0.00 |
| A1 | water seal | 17.50 | 0.26 | -0.19 | 0.29 |
| A2 | warm rinse | 18.08 | 0.18 | -0.04 | 0.26 |
| A3 | oxidation | 18.13 | 0.23 | -0.10 | 0.48 |
| A4 | soaping | 17.30 | 0.10 | -0.37 | 0.77 |
| A5 | cold rinse | 17.79 | 0.16 | -0.15 | 0.25 |

**Table S2.** Influence of processing conditions in soaping on the colour of the dyed sample in terms of CIELab coordinates and total colour difference ΔE between untreated reference and treated sample. Surf = Surfactant (2 g L^-1^ Sandopur RSK, 2 g L^-1^ Na_2_CO_3_; US = ultrasonic treatment).

| Sample | Temp. | Surf | US | Colour coordinate | | | ΔE |
| --- | --- | --- | --- | --- | --- | --- | --- |
|  | °C |  |  | L* | a* | b* |  |
| B0 | - | - | - | 17.14 | -0.03 | -1.25 | 0.00 |
| B1 | 80 | + | - | 17.88 | 0.03 | -0.47 | 1.08 |
| B2 | 80 | - | + | 17.49 | 0.04 | -0.48 | 0.81 |
| B3 | 80 | + | + | 17.66 | -0.08 | -0.63 | 0.85 |
| B4 | 95 | - | - | 18.32 | 0.12 | -0.21 | 1.58 |
| B5 | 95 | + | - | 17.66 | 0.03 | -1.04 | 0.65 |
| B6 | 95 | - | + | 17.64 | 0.07 | -0.85 | 0.34 |
| B7 | 95 | + | + | 17.46 | -0.12 | -1.33 | 0.56 |

**Table S3.** Influence of duration of US treatment in soaping on the colour of the dyed sample in terms of CIELab coordinates and total colour difference ΔE between untreated reference and treated sample.

| Sample | Duration. | Colour coordinate | | | ΔE |
| --- | --- | --- | --- | --- | --- |
|  | s | L* | a* | b* |  |
| C0 | 0 | 19.05 | 0.28 | -0.08 | 0.00 |
| C1 | 30 | 18.14 | 0.24 | -0.20 | 0.92 |
| C2 | 60 | 18.25 | 0.21 | -0.19 | 0.81 |
| C3 | 120 | 18.39 | 0.09 | -0.32 | 0.73 |
| C4 | 300 | 18.87 | 0.34 | -0.04 | 0.19 |
